# Platform-dependent performance of P2A and IRES linkers in multicistronic gene expression

**DOI:** 10.1007/s13258-026-01771-x

**Published:** 2026-04-27

**Authors:** Mingyu Ju, Jinju Han

**Affiliations:** 1 Graduate School of Medical Science and Engineering, Korea Advanced Institute of Science and Technology (KAIST), Daejeon, 34051 Republic of Korea; 2 Graduate School of Stem Cell and Regenerative Biology, KAIST, Daejeon, 34141 Republic of Korea; 3 BioMedical Research Center, KAIST, Daejeon, 34051 Republic of Korea; 4 KAIST Stem Cell Center, KAIST, Daejeon, 34141 Republic of Korea

**Keywords:** mRNA, 2A peptides, Internal Ribosome Entry Site (IRES), N1-methylpseudouridine (m^1^Ψ), Multi-gene expression

## Abstract

**Background:**

Efficient co-expression of multiple genes within individual cells is essential for advances in biotechnology and clinical applications. While 2 A peptides and internal ribosome entry sites (IRESs) are widely used linkers, their performance in synthetic mRNA systems—particularly with modified nucleotides—is insufficiently characterized compared to DNA-based platforms.

**Objective:**

We systematically compared bicistronic gene expression mediated by ribosome skipping peptide P2A and four viral IRES types across plasmid DNA and synthetic mRNA platforms.

**Methods:**

Plasmids were engineered with two open reading frames linked by P2A or four distinct IRES types. mRNAs were generated via in vitro transcription with or without N1-methylpseudouridine (m^1^Ψ) substitution. Expression efficiency was quantified through dual-luciferase assays, using firefly luciferase as a control.

**Results:**

We observed distinct linker-dependent expression patterns between DNA and RNA platforms. P2A generally provided superior overall protein expression in both plasmid DNA and m^1^Ψ –modified mRNA systems, with the exception of unmodified mRNA, in which P2A and the poliovirus (PV; type I) IRES produced comparable downstream gene expression. Full substitution of uridine with m^1^Ψ markedly impaired protein expression driven by all IRES. Notably, even partial m^1^Ψ (25%) was sufficient to disrupt type I IRES activity, whereas P2A constructs displayed a gradual increase in protein output with increasing levels of modification. These results indicate that nucleotide modifications fundamentally alter linker performance.

**Conclusions:**

Our study highlights the importance of linker-dependent effects in the design of RNA-based expression systems, providing insights for optimizing multi-gene expression strategies. These findings may contribute to the development of next-generation mRNA-based therapeutics, including vaccines.

## Introduction

Coordinated expression of multiple genes within individual cells plays a crucial role in advancing biotechnology and clinical applications. For instance, successful cellular reprogramming relies on the expression of multiple transcription factors within a single cell (Takahashi and Yamanaka 2006). Additionally, many diseases, including neurological disorders and cancer, involve complex cellular mechanisms regulated by multiple factors, necessitating multi-gene expression for effective gene therapy (Azzouz et al. 2002; Rayssac et al. 2009; Choi and Kim 2025). To achieve coordinated expression of multiple genes within a single cell, extensive efforts have been made to integrate several genes into a single vector. Although various strategies such as enzymatic proteolysis cleavage factors, multiple promoters and splicing signals have been utilized, the most widely adopted approaches are 2 A peptides and internal ribosome entry sites (IRESs) (Shaimardanova et al. 2019).

The 2 A peptide is an 18- to 22-amino-acid-long viral polypeptide. Since its initial discovery in the foot-and-mouth disease virus, four 2 A peptides—P2A, T2A, F2A, and E2A—have been identified in different viruses (Ryan et al. 1991; Donnelly et al. 1997). All 2 A peptides share a conserved NPGP amino acid sequence, where hydrolysis occurs at the C-terminal of glycine. This ribosome-skipping mechanism enables the synthesis of a new protein starting from proline, ultimately leading to the production of two separate proteins from a single mRNA transcript (Donnelly et al. 2001). Nevertheless, the function of 2 A peptides is not always completely reliable. Occasionally, they produce only the upstream gene product due to ribosome dissociation during hydrolysis. Alternatively, they may generate a fusion protein of the upstream and downstream genes, connected by NPGP, due to hydrolysis failure (Liu et al. 2017). Among the four 2 A peptides, P2A displays the highest ribosome skipping efficiency (Kim et al. 2011).

The IRES is a specific RNA structure that can initiate translation without requiring a cap structure for ribosome interaction. Since the first discovery of IRES in encephalomyocarditis virus (EMCV) and Poliovirus (PV) in 1988, more than 500 IRES structures have been identified in various viruses (Jang et al. 1988; Pelletier and Sonenberg 1988; Zhao et al. 2020). IRES elements have also been found in cellular mRNAs. Although there are a few exceptions, cellular IRESs are generally less conserved in sequence and structure, and display lower translation efficiency compared to viral IRESs (Licursi et al. 2011; Yang and Wang 2019). Due to these limitations, viral IRESs are more commonly used for multi-gene expression. Each viral IRES contain its own structural compactness, which determines the type and number of IRES trans-acting factors (ITAFs) required for translation initiation (Mailliot and Martin 2018). The structural variations serve as the basis for classifying viral IRES into four distinct types.

The mRNA gene delivery platform is gaining significant attention due to its safety. Synthetic mRNAs are non-viral gene delivery molecules with minimal immunogenicity concerns. Moreover, once delivered into cells, synthetic mRNAs are translated in the cytosol and subsequently degraded without integrating into the host genome. While 2 A peptides and IRES elements are applicable for multigene expression in mRNA-based platforms, their use in this context remains largely unstudied, as most research on multicistronic vectors has focused on DNA-based systems. A few independent studies have reported conflicting results regarding the translation efficiency of downstream genes mediated by IRES or 2 A elements (Mizuguchi et al. 2000; Liu et al. 2017; Ko et al. 2019; Lee et al. 2019). However, comparative evaluations of their performance in synthetic mRNA systems remain limited. Given that IRES-mediated translation depends on defined RNA secondary structures, and that chemically modified nucleotides—commonly used to reduce immunogenicity—can alter these structures, it is likely that such modifications may affect IRES function in mRNA-based gene expression systems. Despite this, the impact of modified nucleotides on IRES activity has not been systematically examined.

In this study, we compared bicistronic gene expression mediated by P2A and four distinct classes of viral IRESs across DNA- and mRNA-based gene expression platforms. We demonstrate that the optimal linker for coordinated gene expression is strongly platform-dependent, with fundamentally different performance profiles between plasmid DNA and synthetic mRNA. Moreover, we reveal that modified nucleotide—a feature of therapeutic mRNA—profoundly and differentially impacts linker function. While m^1^Ψ incorporation consistently enhances P2A-mediated translation, it markedly impairs IRES-driven translation in a position- and IRES-type–dependent manner, even at low substitution levels.

## Materials and methods

### Plasmid construction

pcDNA3.1(+) was digested with SacI and ApaI (TAKARA ,1078, 1005) to remove original multiple cloning site (MCS). Complementary oligonucleotides (MG-1 and MG-2) were annealed to generate a double-stranded insert carrying SacI- and ApaI-compatible cohesive ends, and the duplex was ligated into the SacI/ApaI-digested pcDNA3.1(+) vector. The resulting plasmid construct was designated pMG-1. Subsequently, to facilitate IRES insertion, the PstI site was removed by digestion with PstI followed by end blunting using the Klenow fragment (NEB, 2140) and self-ligation. The resulting plasmid construct was designated pMG-2.

To generate an in vitro transcription template, a PCR product was designed in the following order: T7 promoter – human HBB 5’ UTR – Firefly luciferase (FL) – Custom MCS2 – human HBB 3’ UTR – (A_15_C)_3_A_22_ and cloned into the vector. Custom MCS2 comprises seven distinct restriction enzyme sites and was introduced to facilitate subsequent ORF and IRES replacement. To minimize Poly A tail shortening due to the bacterial recombination during cloning, a cytosine was inserted after every 15 adenines within the poly A tail. For template assembly, MG-IVT1 was amplified using primers MG-3 and MG-4 with the pGL3-basic vector as a template, and MG-IVT2 was amplified using primers MG-5 and MG-6 with pGEM T-easy- hHBB 3’ UTR vector (kindly provided by Dr. Kwang-Soo Kim, Harvard Medical School) as a template. MG-IVT1 and MG-IVT2 were then fused by assembly PCR to generate MG-IVT3. The resulting MG-IVT3 PCR product and pMG-2 were digested with HindIII and EcoRI (TAKARA, 1060, 1040), followed by sticky-end ligation to generate pMG-3.

For insertion and replacement of IRES and ORF elements in pMG-3, each component was PCR-amplified using primers (MG-7 to MG-20) containing appropriate flanking restriction sites and cloned into pMG-3. For P2A construct, primer MG-11 and MG-12 was used to maintain an in-frame fusion between upstream and downstream genes without an upstream stop codon. Notably, because the JFH1 and CrPV IRES contain the initiation codon at its 3’ end, primer MG-21 ~ MG-26 was designed to enable direct junction to the ORF2 sequence. All plasmid construct sequences were verified by sanger sequencing. All oligonucleotides used in this study are listed in Table 1.

**Table 1:** Oligonucleotide sequences used in this study

Oligo	5′- Sequence − 3′
MG-1	CTCTGGCTAACTAGAGAACCCACTGCTTACTGGCTTATCGAAATAAGCTTGAATTCTGCAGGGCC
MG-2	CTGCAGAATTCAAGCTTATTTCGATAAGCCAGTAAGCAGTGGGTTCTCTAGTTAGCCAGAGAGCT
MG-3	AGATAAGCTTTAATACGACTCACTATAGGTTAACACATTTGCTTCTGACACAACTGTGTTCACTAGCAACCTCAAATCGATATGGAAGACGCCAAAAACATAAAGAAAG
MG-4	GGATCCCGTCTCGATATCCCGCGGCTGCAGCCCGGGGAGACGTTACACGGCGATCTTTCC
MG-5	GATATCGAGACGGGATCCGCTCGCTTTCTTGCTGTCCAATTTCT
MG-6	AGATGAATTCTTTTTTTTTTTTTTTTTTTTTTGTTTTTTTTTTTTTTTGTTTTTTTTTTTTTTTGTTTTTTTTTTTTTTTACCGGTGCAGCAATGAAAATAAATGTTTTTTATTA
MG-7	CTCAAATCGATATGACTTCGAAAGTTTATGATCCAGAACAAAG
MG-8	CGGCTGCAGCCCGGGGAGACGTTATTGTTCATTTTTGAGAACTCGC
MG-9	CCGCGGGATATCGAGACGATGACTTCGAAAGTTTATGATCCAGAAC
MG-10	GAGCGGATCCTTATTGTTCATTTTTGAGAACTCGC
MG-11	CTCAAATCGATATGGTGAGCAAGGGCGAGG
MG-12	CGGCTGCAGCCCGGGGAGACGTTACTTGTACAGCTCGTCCATGC
MG-13	CGGGATATCGAGACGATGGTGAGCAAGGGCGAGG
MG-14	GAGCGGATCCTTACTTGTACAGCTCGTCCATGCC
MG-15	CGTCTCGATATCCGGTCCAGGATTCTCTTCGACATCTCCGGCTTGTTTCAGCAGAGAGAAGTTTGTTGCCCCGGGGAGACGTTGTTCATTTTTGAGAACTCGCTC
MG-16	GCTCCCGGGGAGACGCTTGTACAGCTCGTCCAT
MG-17	GAACTGCAGCCCCTCTCCCTCCCCC
MG-18	GTCCCGCGGAGGAAAACCACGTCCCCGTG
MG-19	GAACTGCAGTTAAAACAGCTCTGGGGTTG
MG-20	GTCCCGCGGTATGATACAATTGTCTGATTGAAATAACTG
MG-21	GAACTGCAGACCTGCCCCTAATAGGGGC
MG-22	GTTCTGGATCATAAACTTTCGAAGTCATGGTGCACGGTCTACGAGAC
MG-23	CTCGCCCTTGCTCACCATGGTGCACGGTCTACGAGAC
MG-24	GAACTGCAGAAAGCAAAAATGTGATCTTGCTTG
MG-25	GGATCATAAACTTTCGAAGTCATGGTATCTTGAAATGTAGC
MG-26	CTCGCCCTTGCTCACCATGGTATCTTGAAATGTAGC

All sequences are provided in the 5’ to 3’ orientation. F, forward; R, reverse

### In vitro transcription

Plasmid constructs were linearized by digestion with SacI and EcoRI (TAKARA ,1078, 1040), generating a linear DNA template including the sequence from T7 promoter to poly A tail(A_15_C)_3_A_22_ and complete linearization was confirmed by agarose gel electrophoresis. Linearized DNA was purified by gel extraction kit. In vitro transcription was carried out using the T7 Megascript kit (Invitrogen, AMB13345) followed manufacturer’s instruction. CleanCap AG (Trilink, N7113) analogue was added for co-transcriptional capping. For modified mRNA synthesis, N1-methylpseudouridine-5’-Triphosphate (Trilink, N-1081) was used to replace UTP. Reactions were incubated at 37 °C for 6-8 h. DNA templates were removed by DNase I (Invitrogen, AM2238) treatment, followed by RNA purification using acidic phenol-chloroform (Sigma, P1944). Purified mRNA was quantified by microplate reader (Thermo Scientific, Multiskan GO), then stored at -20 °C until use.

### Cell culture

The A549 cells were cultured in a DMEM (Welgene, LM001) with 10% FBS (Gibco, 12483-025) at 37 °C in a humidified incubator with 5% CO_2_. Cells were passaged at ~ 90% confluence using 0.25% Trypsin-EDTA (Welgene, LS015).

### Nucleic acid transfection

A549 cells were seeded one day prior to transfection in a 24-well or 48-well plates at a density of 6.67 × 10^4^ cell/cm^2^. Cells were co-transfected with Renila and firefly luciferase constructs at a 24:1(w/w) ratio using either 250ng total DNA or 500ng total mRNA. For transfection, Lipofectamine 2000 (Thermo Fisher Scientific, 11668027) and transfection-optimizing medium (Welgene, TR004) were used according to the manufacturer’s instruction. Transfection complexes were added to cells as droplet manner.

### Dual luciferase assay

Cells were washed twice with DPBS (Gibco, LB201) in 24 h post transfection. Cells were lysed in Passive Lysis Buffer at room temperature for 15 min, followed by centrifugation at maximum speed for 1 min. Supernatants were transferred to 96-well plate (SPL, 30396). Then, dual luciferase assay was performed using the Dual-luciferase Reporter Assay system reagent (Promega, E1980) (Zhao et al. 2025). Luciferase activities were measured with a multi-plate reader (Thermo Fisher Scientific, Varioskan Flash). Renilla luciferase activity was normalized to firefly luciferase activity.

### Data presentation and statistical analysis

Experiments were performed with at least three independent biological replicates. Data are presented as mean ± SEM. Graphs and statistical analyses were generated using GraphPad Prism (GraphPad software). Statistical analyses were performed using ordinary one-way ANOVA followed by Tukey’s multiple-comparisons test or ordinary two-way ANOVA followed by Šídák’s multiple comparisons test. Statistical significance is indicated in the figures as *p < 0.05; **p < 0.01; ***p < 0.001; and ****p < 0.0001 and non-significant differences are not marked.

## Results

### Linker performance diverges between plasmid DNA and mRNA

**Fig. 1:**
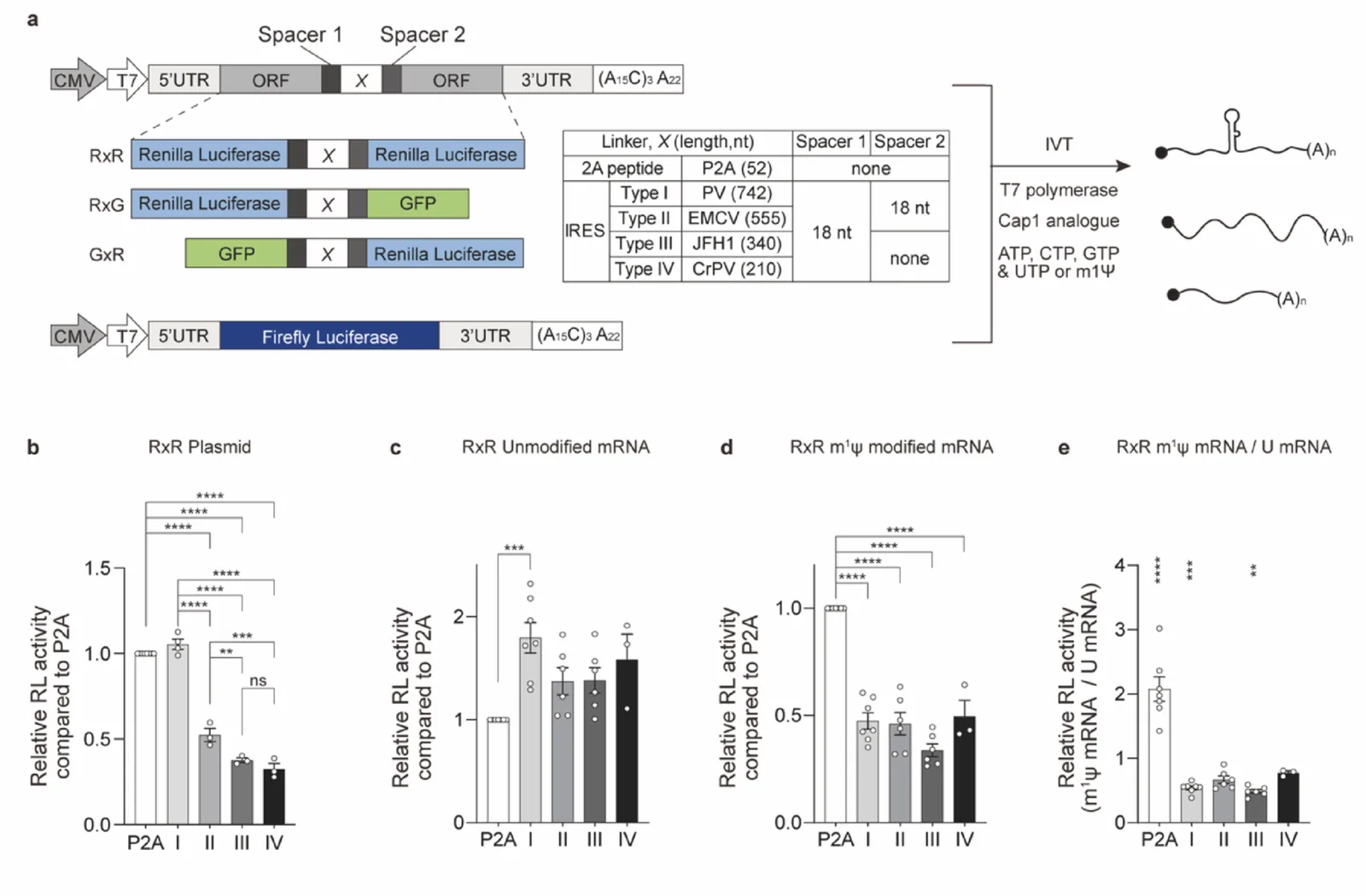
Platform-dependent performance of P2A and IRES linkers in bicistronic gene expression. **a** Schematic overview of the gene constructs used in this study. *Renilla* luciferase (RL) activity was measured to assess linker efficiency, with Firefly luciferase (FL) used as a control. **b**–**d** Relative RL activity of RxR constructs, normalized to the P2A linker. Each dot represents an independent biological replicate, and data are shown as mean ± SEM. Statistical analyses were performed using ordinary one-way ANOVA. **b** Plasmid DNA constructs (P2A, *n* = 8; IRESs, *n* = 3). **c** Unmodified mRNA and **d** m^1^Ψ-modified mRNA. (P2A and type I IRES, *n* = 7; Type II and III IRES, *n* = 6; Type IV IRES, *n* = 3). **e** Relative RL activity of m^1^Ψ-modified mRNA relative to the to the corresponding unmodified counterparts. The same data sets in C and D were used. Data represent mean ± SEM, with each dot indicating an independent biological replicate. Statistical analyses were performed using two-way ANOVA. *p < 0.05; ** *p* < 0.01; *** *p* < 0.001; **** *p* < 0.0001. Unmarked comparisons are not statistically significant

To investigate the efficiency of IRES-mediated multigene expression in both DNA- and mRNA-based platforms, we first constructed a series of plasmids encoding two open reading frames (ORFs) connected by a linker, denoted as ‘*X*’, and flanked by human HBB 5′ and 3′ untranslated regions (UTRs) (Fig. 1a). As linkers, we selected the P2A peptide as a reference and one representative IRES from each of the four distinct types: PV (type I), EMCV (type II), the JFH1 strain of hepatitis C virus (HCV, type III), and cricket paralysis virus (CrPV, type IV). Except for P2A, an identical 18-nt spacer was inserted after the first ORF to minimize potential effects of the spacer on IRES-mediated translation of the downstream ORF. The constructs contain a CMV promoter for transcription in mammalian cells, and a T7 promoter and a synthetic poly(A) sequence composed of (A_15_C)_3_A_22_ for in vitro transcription (Lim et al. 2018; Trepotec et al. 2019). *Renilla* luciferase (RL) or GFP was used as the ORF, and RL activity was quantified using a dual-luciferase assay to evaluate protein expression efficiency. A separate construct encoding firefly luciferase (FL) was used as a control.

DNA plasmid constructs containing tandem RL sequences linked by different linker elements —designated as RL-*X*-RL (RxR)—were first evaluated to determine which linker yielded the highest RL expression (Fig. 1b). RxR plasmids were co-transfected with a FL plasmid into A549 cells, and RL activity was measured 24 h post-transfection and normalized to FL activity. Constructs containing either P2A or type I IRES produced comparable and the highest levels of protein expression among the tested elements. Among the remaining linkers, type II IRES showed relatively higher expression, whereas type III and IV IRES elements resulted in comparable but lower expression levels.

Then, to evaluate whether the same trend was also observed in mRNA-based gene expression constructs that bypass nuclear transcription, RxR mRNAs were synthesized in vitro from the linearized DNA templates with or without N1-methyl-pseudouridine (m^1^Ψ) and co-transfected into A549 cells along with m^1^Ψ-modified FL mRNAs. In unmodified mRNAs, RxR constructs with IRES elements showed a trend toward higher protein expression compared to those with P2A (Fig. 1c). Notably, the unmodified mRNA containing type I IRES displayed significantly greater RL activity compared to the P2A construct. Full substitution of uridine with m^1^Ψ yielded a distinct RL activity profile compared with uridine-containing transcripts (Fig. 1d). Among the constructs containing m^1^Ψ, RL activity was highest in the P2A construct. The P2A construct with m^1^Ψ showed approximately a two-fold increase in RL activity compared to its unmodified, uridine-containing counterpart (Fig. 1e). In contrast, m^1^Ψ reduced RL activity in constructs with type I and type III IRES elements relative to their uridine-containing counterparts, while having no significant effect on constructs with type II or type IV IRES elements, thereby making the overall RL activity of m^1^Ψ-modified IRES constructs comparable to that of the unmodified P2A construct.

### m^1^Ψ differentially influences P2A- and IRES-mediated translation

To determine whether the differences in linker-mediated translation observed in the RxR constructs were driven by the upstream or downstream gene, we examined the impact of gene position relative to the linker. Specifically, we assessed how gene position influences expression efficiency and whether this positional effect differs between DNA- and mRNA-based platforms. To this end, we generated two construct types: RxG, in which RL is positioned upstream of the linker and GFP downstream, and GxR, in which GFP is positioned upstream and RL downstream (Fig. 1a).

**Fig. 2:**
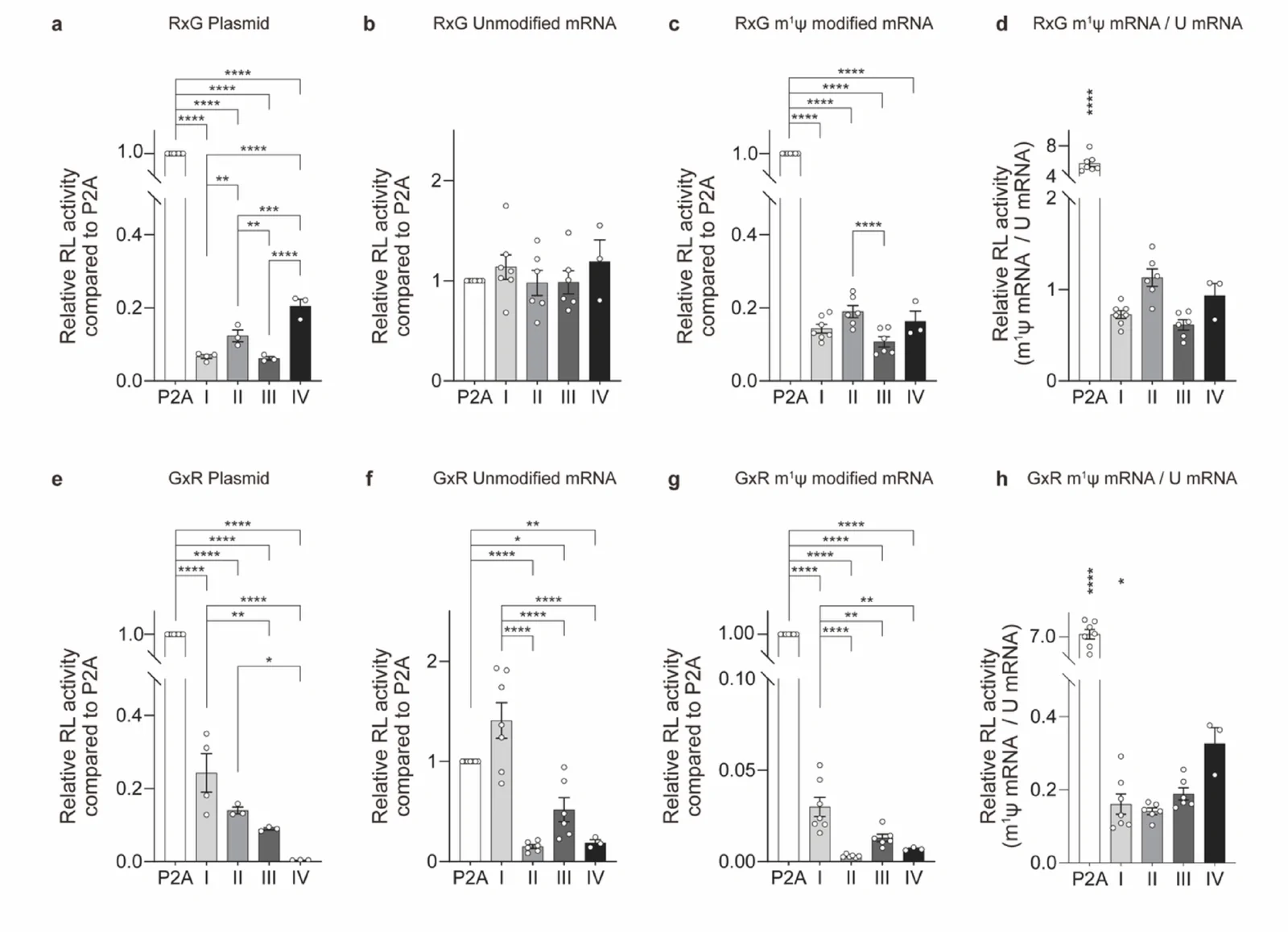
Distinct effects of m^1^Ψ modification on P2A- and IRES-driven RL activity. **a**–**c** Relative RL activity in RxG constructs normalized to the respective P2A in each condition: **a** plasmids, **b** unmodified mRNA, and **c** m^1^Ψ-modified mRNA. **d** RL activity of m^1^Ψ-modified RxG mRNAs, normalized to their corresponding unmodified counterparts. **e**–**h** Analysis of GxR constructs corresponding to panels A-D. Data represent mean ± SEM, with each dot indicating an independent biological replicate. For plasmid experiments (a and e), *n* = 8, 4, 3, 3, and 3 for P2A and type I–IV IRESs, respectively. For mRNA experiments (b–d and f–h), *n* = 7, 7, 6, 6, and 3 for P2A and type I–IV IRESs, respectively. Statistical analyses were performed using ordinary one-way ANOVA (a**–**c and e–g) and two-way ANOVA (d and h). *p < 0.05; ** *p* < 0.01; *** *p* < 0.001; **** *p* < 0.0001. Unmarked comparisons are not statistically significant

Comparable RL activity was initially expected in RxG constructs regardless of the linker used, as RL translation is initiated in a cap-dependent manner. However, in the RxG plasmid constructs, P2A yielded the highest RL activity, whereas all IRES-based linkers achieved less than 20% of that level (Fig. 2a). Among the four IRES linkers, type IV IRES yielded significantly higher RL expression than the others, followed by type II. In the unmodified mRNA platform, RL activity was largely comparable across all linkers (Fig. 2b). In contrast, m^1^Ψ-modified mRNA constructs showed marked differences between P2A and IRES, resembling the plasmid results, as P2A yielded the highest RL activity and all IRES constructs produced less than 20% of that level (Fig. 2c). Interestingly, RL-P2A-GFP mRNA with m^1^Ψ showed six-fold greater RL activity compared to its unmodified counterpart, whereas IRES-linked constructs showed no change (Fig. 2d). We next examined GxR constructs, in which RL was positioned downstream and translated in a linker-dependent manner. As in the RxG constructs, P2A-containing plasmids yielded the highest RL activity compared to constructs with IRESs (Fig. 2e). However, the relative performance of the IRES elements differed between positional context: type I IRES, which had the lowest expression in RxG, produced the highest RL expression in GxR, whereas type IV IRES, which yielded the highest RL expression in RxG, showed the lowest expression in GxR. In unmodified GxR mRNA, the highest RL activity was observed in type I IRES, which was comparable to P2A, whereas type II and IV IRES yielded trivial expression levels (Fig. 2f). In m^1^Ψ-modified mRNA, RL activity from all IRES constructs was negligible (< 5% of P2A) (Fig. 2g). However, among IRES constructs in m^1^Ψ-modified mRNA, the type I IRES showed significantly higher RL activity than those of other IRES types. Notably, fully m^1^Ψ substitution in the P2A-containing mRNA resulted in a ~seven-fold increase in RL activity compared to its unmodified counterpart (Fig. 2h).

### Low-level m^1^Ψ incorporation impairs IRES-mediated translation

Given that the translation initiation function of the IRES hinges on structural integrity, the bulky methyl group of m^1^Ψ may interrupt IRES structure. Hence, we tested whether lowering the proportion of m^1^Ψ could preserve IRES function. To this end, the molar ratio between m^1^Ψ and uridine was adjusted at the stage of in vitro transcription. On the basis of their contrasting activity patterns under m^1^Ψ modification, P2A and type I IRES were selected for further analysis.

**Fig. 3:**
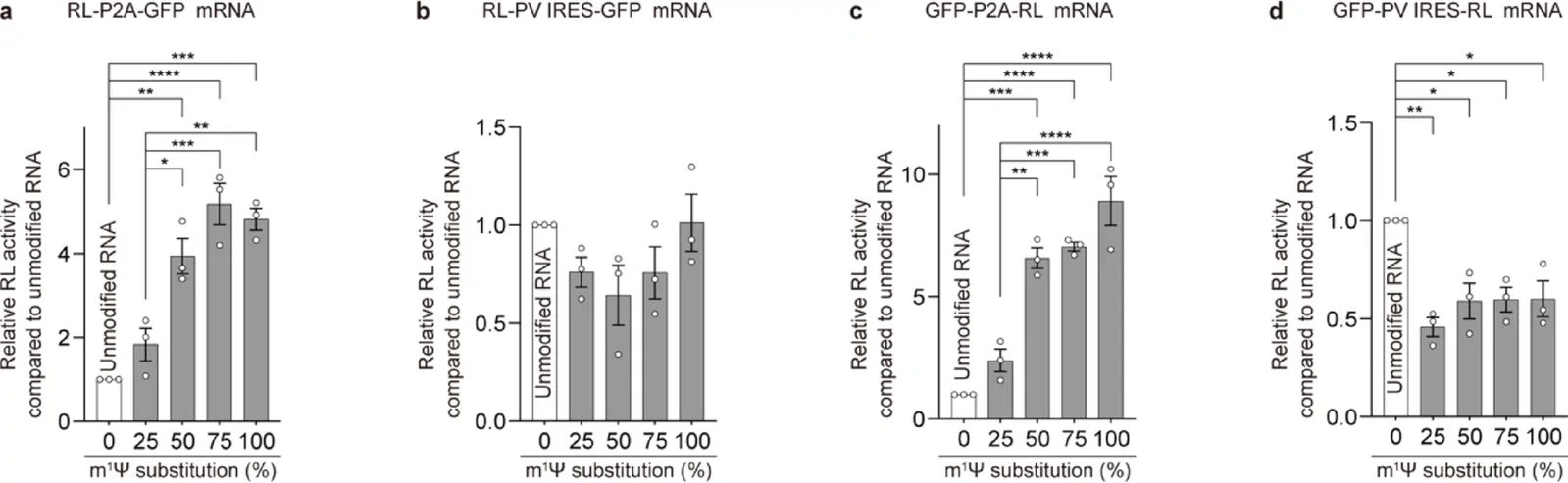
Differential m^1^Ψ dose-response of P2A versus PV IRES in RL activity. Relative RL activity of **a** RL–P2A–GFP, **b** RL–PV IRES–GFP, **c** GFP–P2A–RL, and **d** GFP–PV IRES–RL constructs at different m^1^Ψ incorporation ratios, normalized to unmodified mRNA (0% m^1^Ψ). Data represent the mean ± SEM, with each dot indicating an independent biological replicate, *n* = 3. All statistical analyses were performed using ordinary one-way ANOVA. *p < 0.05; ** *p* < 0.01; *** *p* < 0.001; **** *p* < 0.0001. Unmarked comparisons are not statistically significant

In the RL-P2A-GFP mRNA, a higher m^1^Ψ incorporation ratio led to a corresponding increase in RL activity (Fig. 3a). In contrast, there was no significant change in RL activity in type I IRES constructs, regardless of m^1^Ψ incorporation levels (Fig. 3b). A similar pattern was observed in GxR mRNA constructs bearing P2A linkers, where RL activity increased in near proportion to m^1^Ψ incorporation ratio (Fig. 3c). However, the type I IRES construct exhibited a significant decrease in RL activity with only 25% m^1^Ψ incorporation (Fig. 3d). For both P2A-containing constructs, at least 50% of m^1^Ψ incorporation was required to achieve a clear increase in RL activity compared to the unmodified mRNA (0% m^1^Ψ). In contrast, type I IRES constructs maintained unchanged translation of the upstream gene even with increasing m^1^Ψ incorporation, whereas translation of the downstream gene was significantly impaired at incorporation levels as low as 25%.

## Discussion

Our study demonstrates that the optimal linker strategy for multicistronic gene expression is not universally conserved but instead depends strongly on the expression platform and RNA modification context. By systematically comparing P2A- and IRES-based strategies across plasmid DNA and synthetic mRNA platforms, we show that P2A-mediated constructs generally provide superior overall protein expression in both plasmid DNA and m^1^Ψ-modified mRNA systems. In contrast, in unmodified uridine mRNA, upstream gene expression was largely insensitive to linker choice, while downstream gene expression was comparable between P2A- and PV-IRES (type I)-based constructs. These findings highlight a platform- and modification-dependent hierarchy of linker performance that should be considered when designing multicistronic mRNA systems.

We compared P2A-mediated multigene expression with several IRES-mediated systems and initially expected similar levels of upstream ORF protein expression, as translation of the upstream ORF is cap-dependent in all constructs. However, in the RxG constructs, RL expression was unexpectedly higher in the P2A construct than in the IRES-containing constructs, despite RL being translated via a cap-dependent mechanism (Fig. 2a and c). In the P2A-mediated system, translation proceeds through a single open reading frame with a single termination codon located at the end of the downstream ORF. In contrast, in the IRES-mediated system, the upstream ORF contains its own termination codon, after which the IRES and downstream ORF may function analogously to a 3′ UTR. It is well established that the length, sequence motifs, and structural features of the 3′ UTR can influence translation efficiency of the ORF (Tanguay and Gallie 1996; Lai et al. 2022; West et al. 2025). These features may therefore contribute to the reduced upstream RL expression observed in the IRES-containing constructs, even though translation initiation occurs via a cap-dependent mechanism in all cases.

Unlike plasmid DNA and m^1^Ψ-modified mRNAs, unmodified mRNA produced similar levels of upstream ORF expression across all constructs (Fig. 2b). This effect is likely due to reduced translational efficiency of the P2A construct rather than alterations in translation of IRES constructs (Fig. 2d). m^1^Ψ enhances protein production by stabilizing mRNA and increasing translational efficiency (Kariko et al. 2008; Andries et al. 2015; Parr et al. 2020), while also mitigating global translational suppression by avoiding innate immune activation (Svitkin et al. 2017; Acevedo et al. 2018). In contrast, unmodified mRNAs are more susceptible to immune activation, which can broadly suppress cap-dependent translation. Therefore, cap-dependent translation of RL in the IRES constructs would also be expected to decrease under these conditions. However, the comparable levels of upstream ORF expression across IRES constructs regardless of m^1^Ψ (Fig. 2d) suggest that additional mechanisms may be involved. Although this requires further investigation, one possibility is that IRES elements, such as the PV IRES, may enhance translation of the upstream ORF (Junemann et al. 2007), thereby compensating for reduced global translation efficiency.

Downstream ORF protein expression, translated via IRES, is affected by m^1^Ψ modification. RL protein produced downstream of all IRES elements showed a trend toward reduction, reaching statistical significance only for the PV IRES (Fig. 2f-h). Analysis of varying molar fractions of m^1^Ψ further underscored this divergence. Translation efficiency of P2A constructs increased gradually with higher m^1^Ψ incorporation, whereas PV IRES–driven translation exhibited an abrupt loss of activity that was already evident at the minimal substitution level tested (25%) (Fig. 3d). The presence of a methyl group at the N1 position may influence local base interactions and RNA folding (Mauger et al. 2019; Dutta et al. 2025). Previous studies have reported that m^1^Ψ can alter the RNA structural landscape (Mauger et al. 2019). Given that IRES activity is highly dependent on its secondary structure, complete substitution of uridine with m^1^Ψ may be particularly detrimental to IRES-mediated translation. However, IRES function depends not only on RNA structural integrity but also on protein–IRES interactions. As m^1^Ψ has been reported to interfere with protein–RNA interactions (Andries et al. 2015; Kim et al. 2025), a graded reduction in translation would be expected if disrupted protein binding were the primary mechanism. However, the absence of such a graded response argues against disrupted protein–IRES interactions as the sole mechanism. Focusing specifically on the PV IRES used in this study, it is notable that picornaviral IRES elements generally harbor polypyrimidine-rich regions that interact with polypyrimidine tract-binding protein 1 (PTBP1) and influence binding of translation initiation factor 4B, eIF4B (10.1128/jvi.76.5.2113-2122.2002). The PV IRES has been reported to show strong dependence on PTBP1 for maintaining its structural integrity (Nishimura et al. 2008). It is therefore possible that m^1^Ψ modification affects RNA conformation and RNA-protein (including PTBP1) interactions. Thus, the pronounced sensitivity of type I IRES to m^1^Ψ may reflect its reliance on structurally and protein interaction-dependent regulatory mechanisms. The substantial impairment observed for the compact, ITAF-independent type IV CrPV IRES is consistent with the possibility that m^1^Ψ substitution more broadly perturbs core IRES RNA structural features required for internal initiation. Such structural alterations could also affect mRNA stability, potentially contributing to the lack of increased RL expression in RxG constructs containing m^1^Ψ.

Although IRES-based linkers were incompatible with m^1^Ψ-modified mRNA in this context, they supported translation efficiencies comparable to P2A in unmodified mRNAs. While unmodified mRNA is generally avoided in therapeutic applications due to immunogenicity, alternative platforms such as circular RNA—characterized by immune evasion and robust translation—may provide contexts in which IRES-based strategies remain advantageous. Indeed, recent studies have identified type I IRES elements with high translational activity in circular RNA systems (Chen et al. 2023). In addition, in hypo-responsive immune environments such as induced pluripotent stem cells, unmodified mRNA–IRES constructs may offer practical benefits (Guo et al. 2015).

Clinical demand for coordinated multi-gene expression is substantial (Shaimardanova et al. 2019), as exemplified by gene therapy (Azzouz et al. 2002; Rayssac et al. 2009) and cellular reprogramming strategies (Carey et al. 2009; Warlich et al. 2011) that require the simultaneous expression of multiple factors. While 2 A-based mRNA constructs offer advantages in translation efficiency and stoichiometric control (Lee et al. 2019), their application can be limited by residual amino acid sequences that may interfere with proper protein processing or localization (Hadpech et al. 2018). In such cases, IRES-based systems may provide a useful alternative. However, several considerations remain for their broader application. This study examined only a limited number of linker elements and reporter genes, which may constrain generalizability. In addition, because therapeutic mRNAs are typically delivered using lipid nanoparticles (LNPs), IRES performance should be evaluated in delivery-relevant contexts and across multiple cell types beyond A549 cells, as IRES activity is known to exhibit cell-type specificity. This specificity likely reflects differences in the expression of eukaryotic initiation factors and ITAFs, as well as modulation by microRNAs reported to stabilize IRES structures (Schult et al. 2018). Future studies employing systematic screening approaches may help identify linker elements optimized for specific cell types and delivery platforms. Despite these limitations, our study demonstrates that linker choice and RNA modification jointly shape translational outcomes, providing practical guidance for the rational design of multicistronic mRNA constructs and informing the development of optimized mRNA-based therapeutic platforms.

## Data Availability

The data that support the findings of this study are available from the corresponding author (J.H.), upon reasonable request.
