# Correction: Platform-dependent performance of P2A and IRES linkers in multicistronic gene expression

**DOI:** 10.1007/s13258-026-01799-z

**Published:** 2026-08-25

**Authors:** Mingyu Ju, Jinju Han

**Affiliations:** 1 Graduate School of Medical Science and Engineering, Korea Advanced Institute of Science and Technology (KAIST), Daejeon, 34051 Republic of Korea; 2 Graduate School of Stem Cell and Regenerative Biology, KAIST, Daejeon, 34141 Republic of Korea; 3 BioMedical Research Center, KAIST, Daejeon, 34051 Republic of Korea; 4 KAIST Stem Cell Center, KAIST, Daejeon, 34141 Republic of Korea

**Correction to: Genes & Genomics (2026) 48:1007–1016** **https://doi.org/10.1007/s13258-026-01771-x**

In the original version of the article, the authors identified errors in the reference list and funding information.

Reference correction

In the main text, the citation currently appears as:

(https://doi.org/10.1128/jvi.76.5.2113-2122.2002)

This should be corrected to:

(Ochs et al., 2002)

In addition, the following reference should be added to the Reference section between Nishimura et al. (2008) and Parr et al. (2020):

Ochs K, Saleh L, Bassili G, Sonntag VH, Zeller A, Niepmann M (2002) Interaction of translation initiation factor eIF4B with the poliovirus internal ribosome entry site. Journal of Virology 76:2113–2122.

Funding correction

The Funding section currently states:

“RS-2024-00398786 to JH, , RS-2025-25438316 to MJ”

This should read:

“RS-2024-00398786 to J.H., RS-2025-25438316 to M.J.”

In addition, the text:

“(RS-2025-02223058 to JH)”

should read:

“(RS-2025-02223058 to J.H.)”

This has been corrected in this article.

